# Stress and Burnout Among Anesthesia Technologists, Technicians, and Trainees: A Cross-Sectional Study in a Tertiary Hospital in Saudi Arabia

**DOI:** 10.3390/healthcare13020119

**Published:** 2025-01-09

**Authors:** Mohmad H. Alsabani, Fay Aljohani, Ghaid Rakan Alkathiri, Judy Saad Alkhonain, Lama Aljuhani, Shahad Alanazi, Lafi H. Olayan, Turki Aljuhani, Faraj K. Alenezi, Mohammed K. Al Harbi

**Affiliations:** 1 Anesthesia Technology Department, College of Applied Medical Sciences, King Saud bin Abdulaziz University for Health Sciences, Riyadh 11481, Saudi Arabiaghaidalkathiri@gmail.com (G.R.A.); 421210035@ksau-hs.edu.sa (J.S.A.); 421210253@ksau-hs.edu.sa (L.A.); shahadalanzi77@gmail.com (S.A.); olayanl@ksau-hs.edu.sa (L.H.O.);; 2 King Abdullah International Medical Research Centre, Riyadh 11481, Saudi Arabia; juhanit@ksau-hs.edu.sa (T.A.); harbimk@ngha.med.sa (M.K.A.H.); 3 Department of Occupational Therapy, College of Applied Medical Sciences, King Saud bin Abdulaziz University for Health Sciences, Riyadh 11481, Saudi Arabia; 4 Department of Anesthesia, Ministry of National Guard Health Affairs, Riyadh 11426, Saudi Arabia; 5 College of Medicine, King Saud bin Abdulaziz University for Health Sciences, Riyadh 11481, Saudi Arabia

**Keywords:** burnout, stress, anesthesia technologist, anesthesia technicians, trainees, perioperative healthcare providers, emotional exhaustion, depersonalization, personal accomplishment

## Abstract

**Background/Objectives:** Occupational burnout poses a significant burden to healthcare personnel, institutions, and service users. Anesthesia technologists and technicians work in the shadow of the perioperative team, and a lack of attention to anesthesia support personnel may pose a significant risk to their wellbeing and the quality of care delivered. To date, only a few studies have investigated the prevalence of burnout among anesthesia technologists and technicians worldwide and in Saudi Arabia. Thus, the aim of this cross-sectional study was to assess the prevalence and contributing factors to burnout among anesthesia technologists and technicians in a single tertiary hospital in Saudi Arabia. **Methods:** The study utilized the Maslach Burnout Inventory-Human Services Survey (MBI-HSS) to assess burnout and a 10-point scale to assess stress levels. The MBI-HSS inventory consists of three subscales: emotional exhaustion (EE), depersonalization (DP), and personal accomplishment (PA). Univariate and multivariate linear regression analyses were used to identify correlates of each burnout subscale. Gender was included in the multivariable regression analysis in addition to significant variables from univariable analysis. **Results:** A total of 89 participants completed the survey. Based on each subscale of the MBI-HSS, more than 60% of the participants reported high to moderate EE, and more than half reported high to moderate DP. For PA, only 25.8% of participants reported low PA. We found that age (β = −0.58, 95% CI: −0.95, −0.20; *p* = 0.003) and stress (β = 3.3, 95% CI: 2.1, 4.5; *p* < 0.001) were independently associated with EE. In addition, night shift (β = 3.3, 95% CI: 0.44, 6.1; *p* = 0.024) and stress (β = 0.73, 95% CI: 0.13, 1.3; *p* = 0.017) were independently associated with DP. Independent factors for PA were identified including night shifts (β = 6.6, 95% CI: 1.4, 12; *p* = 0.014) and stress levels (β = −1.3, 95% CI: −2.4, −0.12; *p* = 0.03). **Conclusions:** This research underscores the alarmingly high prevalence of burnout and the strong link between elevated EE and DP rates and workplace stress, emphasizing the necessity to identify and mitigate these stressors. It is therefore crucial to evaluate the effectiveness of the current wellbeing and mental health initiatives and programs in Saudi Arabia to ensure that they address evolving challenges and the overall mental health of healthcare personnel.

## 1. Introduction

Freudenberg [1] first defined the term “burnout” and Maslach et al. [2] developed it as a chronic stress condition marked by extreme emotional, physical, and mental tiredness. Burnout is an occupational phenomenon characterized by low personal accomplishments (PA), emotional exhaustion (EE), and depersonalization (DP) [3]. Emotional exhaustion, the most obvious manifestation of burnout syndrome, refers to feeling emotionally worn out, tired, and psychologically drained. Depersonalization involves disengagement from work and skepticism by minimizing one’s involvement in work. Reduced personal accomplishment is characterized by a lack of achievement and a sense of frustration within the job, resulting from a loss of confidence in one’s ability and a sense of inadequacy [4].

Burnout is recognized as a critical component of general health and has been included in the 11th Revision of the International Classification of Diseases (ICD-11). Numerous factors contribute to the risk of developing burnout at both the individual and organizational level. On the individual side, for example, younger age and female gender, negative coping strategies are associated with higher burnout risk [5,6]. On the other hand, organization-related factors such as the imbalance between life and work and unclear job expectations could increase the risk of burnout [7]. Affected healthcare workers often experience numerous signs of burnout. These include a sudden change in personality, irritability, lack of concentration, physical fatigue, and anxiety [8]. In addition, burnout can alter personal function, causing both mental illness such as anxiety, sleep disturbances, and depression, and medical complications such as high blood pressure, muscle tension, and headaches [8,9].

Healthcare providers are generally at a higher risk due to multiple factors, including the following: (1) increased productivity requirements; (2) decreased reimbursements with increased regulation; (3) less time with patients; (4) requirement of rapidly expanding medical knowledge (increased number of hours for continuing training with less to no allocation time for training); (5) difficulty balancing personal and professional life because of extensive work hours and requirement for night calls. All these factors contributed to the high prevalence of burnout among healthcare providers.

Occupational burnout poses a serious issue as it may lead to a number of detrimental outcomes, including the following: poor patient care [10], poor patient satisfaction of care provided [11], strained professional relationships, drug misuse [12,13], and frequent medical errors [14,15]. The prevalence of burnout differs between medical specialties. Different studies suggest that anesthesia and critical care providers often experience a higher risk of burnout compared to other medical specialties [5,16]. Burnout etiology can be attributed to multifactorial reasons, with internal and external factors that may lead to burnout. The most inflectional factors are the following: job demand control, effort–reward imbalance, and organizational injustice [17].

Job stress is a major concern for many healthcare providers. It represents the continual emotional burden due to increasing job requirements. The work environment or the employee’s personality and lifestyle can trigger this response. The absence of breaks during the workday, burdensome workload, and high-demanding environment can lead to job stress [18]. Stress and burnout are interconnected. McManus and colleagues established the causal link between stress and burnout among UK doctors and found that EE is critical to the stress–burnout cycle, in which both EE and stress influence each other bidirectionally [19]. On the other hand, DP decreases stress, possibly through ego-defense mechanisms, and PA increases stress, directly or indirectly, through EE. Multiple studies found that job stress is a concerning issue for anesthesiologists. A more recent study in the U.S. indicated that the demand for anesthesiologists increased, and staff shortages following COVID-19 contributed to heightened job stress. This stress has resulted in burnout and an increased likelihood of staff leaving their positions within the next two years [20].

Although there have been previously published studies on burnout syndrome and job stress among anesthesia providers, and despite the widespread and well-understood nature of the phenomenon, there is a lack of studies specifically examining the situation among anesthesia technologists, technicians, and trainees in Saudi Arabia. To date, there is only one recent study conducted by Almodibeg, which included a relatively small sample size of 19 anesthesia technicians. In addition, the Saudi Commission for Health Specialties released a guidance framework in 2022 to help training centers build their internal wellbeing systems, acknowledging the magnitude of occupational burnout as a major threat to the healthcare delivery system. Furthermore, in 2019, Saudi Arabia launched the National Center for Mental Health Promotion to enhance mental health across various workplace settings. Given these advancements, this could reveal critical insights and highlight areas that may need further intervention. Thus, this study aimed to (i) identify the prevalence of burnout among anesthesia technologists, technicians, and trainees in a tertiary hospital in Saudi Arabia, (ii) investigate factors that contribute to burnout among anesthesia technologists and technicians, and (iii) examine the contribution of job stress to burnout among the studied population. Accordingly, we hypothesized that a significant proportion of anesthesia technologists/technicians and trainees experience moderate to high levels of burnout and occupational stress serves as a main source of burnout.

## 2. Materials and Methods

### 2.1. Study Design and Eligibility Criteria

This study employed a quantitative, cross-sectional survey study design utilizing an online platform (Microsoft Forms) to explore the stress level and occupational burnout among anesthesia support healthcare personnel in a tertiary hospital in Riyadh, Saudi Arabia. The study population includes Saudi and non-Saudi anesthesia technologists, technicians, and anesthesia technology interns/trainees. All other healthcare providers and field training anesthesia technology students were excluded from the study.

### 2.2. Ethical Approval

The study was approved by the institutional review board of the King Abdullah International Medical Research Center (KAIMRC) (SP23R/047/04), from 19 June 2023 to 19 June 2024, in Riyadh, Saudi Arabia. Furthermore, a license to reproduce 100 copies of the Maslach Burnout Inventory-Human Services Survey (MBI-HSS) for medical personnel was obtained from Mind Garden. Informed consent was obtained prior to anonymously answering the survey.

### 2.3. Participants and Data Collection

Anesthesia technologists, technicians, and trainees in the field working at National Guard Health Affairs-affiliated hospitals in Riyadh, Saudi Arabia, were invited to participate in the study through a non-random convenience sampling method. Anesthesia technologists and technicians are non-physician, licensed healthcare professionals who assist and provide critical support to the anesthesiologist during anesthesia care. Their functional responsibilities in clinical settings are the same. However, they were differentiated based on their academic qualifications, job titles, and professional classifications. In the context of Saudi Arabia, anesthesia technicians typically hold a diploma, whereas anesthesia technologists generally have more advanced education, such as a bachelor’s degree. Anesthesia technology trainees in our study refer to individuals enrolled in anesthesia technology training programs who have graduated with an undergraduate certificate but have not yet completed the required one-year clinical training necessary for licensure. The eligible participants were invited to participate in this study through in-person field visits to the operating theater in the hospital by researchers. In addition, the participants accessed the survey through an iPad provided by the researchers during data collection. Participation was entirely voluntary, and informed consent was secured prior to the completion of the study questionnaire. In addition, the survey opens with an introduction letter that clearly outlines the aims of the study, the ability to withdraw from it at any time, the name and contact details of the lead researcher for questions, and an assurance statement advising that the information gathered will remain confidential and anonymous. Surveys that were not fully completed were excluded from the final analysis. The study survey was designed to be completed in 3–5 min. All responses were finalized, saved on a web server, and transferred to an Excel sheet for future reference. Due to the nature of this exploratory study, there was no need for sample size calculations.

### 2.4. Instrument

The survey consisted of three parts. The first part included questions regarding the demographic characteristics of the participants, including gender, age, marital status, smoking, number of cases attended per week, frequency of challenging cases, income, and night shifts. These factors were identified from the relevant literature [5,20,21,22]. The second part was a 10-point self-assessment scale to measure the stress, ranging from 0—no stress at all to 10—extreme stress. The single-item measure of stress has been shown to provide meaningful results in previous studies [23,24]. The third part was an English version of the MBI-HSS, which is a scale for measuring burnout among human services workers. This tool has gained worldwide acceptance for its ability to measure burnout, as it was the first to exclusively score burnout among healthcare providers. The reliability and validity of the MBI-HSS have been demonstrated across diverse populations and settings, including anesthesia practitioners [25]. MBI-HSS consists of 22 items that evaluate the three burnout dimensions: low PA with 8 items, EE with 9 items, and DP with 5 items. The respondents were asked to report their specific feelings about work on a 7-point scale, from 0—never; 1—once or twice a year or less; 2—once a month or less; 3—a few times a month; 4—once a week; 5—a few times a week; and 6—every day. Each subscale in the MBI-HSS inventory is assessed using a series of questions. The final score for each subscale for every participant is calculated by adding the scores for items specific to each subscale. Burnout levels were measured by adding the items’ responses for each subscale to get the total score on each subscale. Then, the total score was divided by the number of items measured by each subscale to get the average score. The levels of burnout have been categorized as suggested by Maslach and Jackson [4] as follows: high burnout for EE ≥ 27, ≥10 for DP, and ≤33 for PA. For moderate burnout, the score ranges from 19 to 26 for EE, 6 to 9 for DP, and 34 to 39 for PA. Finally, low burnout has a score of ≤18 for EE, ≤5 for DP, and ≥40 for PA.

### 2.5. Statistical Analysis

Descriptive statistics were used to summarize the data. Categorical data were summarized using frequencies and percentages, and the median and interquartile range (IQR) for continuous data. Inferential statistics, including the Mann–Whitney U test, was used to compare the sociodemographic characteristics, stress level, and total scores of all the burnout subscales between anesthesia technologists/technicians and trainees. The Kruskal–Wallis test was used to examine the association between burnout and stress levels among the entire sample. Univariable and multivariable linear regression analyses were conducted to identify the associations of burnout-related subscales with sociodemographic variables and stress levels, with the results reported as Beta and 95% confidence intervals (CIs). Variables with a *p*-value < 0.05 in univariable analysis were included in a backward stepwise multivariable linear regression model to determine independent predictors of burnout. The multivariate model incorporated factors that showed statistical significance in univariate analysis and gender as a relevant variable highlighted in the literature. A two-sided *p*-value < 0.05 was considered statistically significant. Statistical analyses were performed using IBM SPSS Statistics for Mac (version 27.0) and R statistical software (version 4.2.0).

## 3. Results

### 3.1. Characteristics of the Respondents

A total of 89 anesthesia technologists (n = 54), technicians (n = 4), and trainees (n = 31) completed the survey, resulting in a response rate of 91.75%. As shown in Table 1, the majority of respondents were male (n = 68, 76.4%), single (n = 71, 79.8%), and smokers (n = 32, 36%). The median age of respondents was 24 years (IQR: 4 years). Regarding income, 37.1% earned less than SAR 6000, 27% earned between SAR 9000 and 12,000, and 22.5% earned between SAR 6000 and 9000. A total of 9% earned between SAR 12,000 and 16,000, and 4.5% earned more than SAR 16,000. The median number of cases per week was 4 (IQR: 2). In terms of the frequency of challenging cases, 36% reported 2–3 challenging cases per week, 31.5% encountered 1 per week, and 16.9% faced a challenging case once per month. About 9% dealt with challenging cases almost every day, while 6.7% rarely faced challenging cases. Additionally, 46.1% worked night shifts. Table 1 also shows that there were statistically significant differences in age, gender, smoking, and income between the anesthesia technologists/technicians and trainees. However, no statistical difference was found between both groups in the number of cases per week and the frequency of challenging cases. Similarly, there were no statistically significant differences between the anesthesia technologists/technicians and trainees in the total score of all three burnout subscales and stress levels. Both groups reported moderate EE, DP, and low PA.

### 3.2. Prevalence of Burnout

Table 2 shows the prevalence of burnout across the subscales. High EE was reported by 38.2% of respondents, indicating significant feelings of fatigue and depletion, while moderate EE was reported by 23.6%, and low EE by 38.2%. DP was high in 28.1%, reflecting notable detachment or cynicism, while moderate and low DP were reported by 28.1% and 43.8% of respondents, respectively. Regarding PA, more than half of the respondents reported high levels, while moderate and low levels of PA were reported by 15.7% and 25.8%, respectively.

### 3.3. Association Between Burnout and Job Stress

Table 3 presents the association between burnout and job stress levels. Emotional exhaustion was significantly associated with job stress (*p* < 0.001). Respondents with high emotional exhaustion reported a median stress level of 8.0 (IQR: 2.0), those with moderate exhaustion had a median of 6.0 (IQR: 3.0), and those with low exhaustion had a median of 5.5 (IQR: 4.0). A significant association was also observed between depersonalization and job stress (*p* = 0.015). Respondents with high depersonalization had a median stress level of 8.0 (IQR: 2.0), while those with moderate and low depersonalization had median stress levels of 6.0 (IQR: 3.0 and 5.0, respectively). Additionally, personal accomplishment was significantly associated with job stress (*p* = 0.048). Respondents with high personal accomplishment reported a median stress level of 8.0 (IQR: 3.0), those with moderate accomplishment had a median of 7.0 (IQR: 3.25), and those with low accomplishment had a median of 6.0 (IQR: 4.0).

### 3.4. Factors Associated with Emotional Exhaustion

Univariate and multivariate analyses of factors associated with emotional exhaustion are shown in Table 4. In the univariate analysis, emotional exhaustion was significantly associated with age (β = −0.50, 95% CI: −0.94, −0.05; *p* = 0.029), frequency of challenging cases (once per week: β = 2.0, 95% CI: −6.2, 10; *p* = 0.010; 2–3 times per week: β = 8.0, 95% CI: 0.09, 16; *p* = 0.010; almost every day: β = −8.9, 95% CI: −20, 2.2; *p* = 0.010; almost none: β = −1.6, 95% CI: −14, 11; *p* = 0.010), and stress level (β = 3.3, 95% CI: 2.1, 4.4; *p* < 0.001). In the multivariate analysis, age (β = −0.58, 95% CI: −0.95, −0.20; *p* = 0.003) and stress level (β = 3.3, 95% CI: 2.1, 4.5; *p* < 0.001) remained independently associated with emotional exhaustion (Table 4).

### 3.5. Factors Associated with Depersonalization

Univariate and multivariate analyses of factors associated with depersonalization are shown in Table 5. In the univariate analysis, stress levels (β = 0.74, 95% CI: 0.13–1.4; *p* = 0.018), night shifts (β = 3.3, 95% CI: 0.61–5.9; *p* = 0.016), and smoking (β = 3.0, 95% CI: 0.26–5.8; *p* = 0.032) were associated with depersonalization. In the multivariate analysis, stress levels (β = 0.73, 95% CI: 0.13–1.3; *p* = 0.017) and night shifts (β = 3.3, 95% CI: 0.44–6.1; *p* = 0.024) remained independently associated with depersonalization (Table 5).

### 3.6. Factors Associated with Personal Accomplishment

Univariate and multivariate analyses of factors associated with personal accomplishment are shown in Table 6. Factors associated with personal accomplishment in the univariate analysis included the night shifts (β = 5.0, 95% CI: 0.04, 10; *p* = 0.048) and higher stress levels (β = −1.2, 95% CI: −2.3, −0.03; *p* = 0.043). The multivariate analysis identified working night shifts (β = 6.6, 95% CI: 1.4, 12; *p* = 0.014) and stress levels (β = −1.3, 95% CI:−2.4, −0.12; *p* = 0.03) as independent predictors of personal accomplishment (Table 6).

## 4. Discussion

Burnout and job stress are growing concerns within the healthcare sector, not only impacting the wellbeing of healthcare professionals but also having potential adverse implications for patient safety and quality of care. This study examined the prevalence of burnout among ATs and further looked at factors that are associated with all burnout subscales (EE, DP, and PA). Our findings showed that over half of the respondents reported moderate to high levels of EE and DP, and approximately 25% of participants reported low PA. Our study also identified specific factors that contribute to each subscale of the burnout inventory. Notably, age and stress were independent factors of EE, while night shifts and stress were independently associated with higher levels of DP.

Recent developments aimed at enhancing patient safety, along with changes in healthcare policies, have led to increasingly challenging and high-pressure work settings that subject providers in high-demand settings to a variety of stressors [26,27,28]. Concerns regarding the effects of burnout on anesthesia providers and their patients have prompted practitioners and researchers to shed light on this issue [29]. Our study confirms the considerable prevalence of burnout and stress among anesthesia technologists, technicians, and trainees. We found that a high proportion of respondents indicated high levels of EE (38.1%) and DP (28.1%), whereas 25.8% exhibited low levels of PA. This aligns with previous studies, including a cross-sectional study in Kosovo that surveyed 154 anesthesiologists and anesthesia technicians [30]. Around one-third of the participants showed significant levels of burnout. In Saudi Arabia, the prevalence of occupational burnout among anesthesia technicians was 29%, with more than 40% of participants reporting emotional exhaustion, while more than half reported depersonalization, and 76.5% reported a lack of personal accomplishment (76.5%) [31]. However, the study’s limitations, such as the small sample size and gender bias, restrict the generalizability of their findings.

Despite the scarcity of published studies on anesthesia technologists and/or technicians, similar studies in the literature investigated burnout among other members of the anesthesia team. For example, Lea et al. (2022) explored burnout among Certified Registered Nurse Anesthetists (CRNAs) in the context of the COVID-19 pandemic [32]. The authors reported significant levels of occupational burnout (38.7%) during the COVID-19 pandemic, with around 80% experiencing considerable disengagement alongside varying degrees of exhaustion. Furthermore, Chipas and McKenna (2011) demonstrated among CRNAs and student registered nurse anesthetists (SRNAs) significant levels of emotional exhaustion, elevated stress levels, and a decline in career/workplace satisfaction among SRNAs in particular [23]. A significant number of participants reported stress-related symptoms such as agitation and trivial annoyances, highlighting the fact that burnout impacts not only mental health. In our study, anesthesia technology trainees reported burnout subscale scores and stress levels comparable to those of licensed anesthesia technologists and technicians. Both groups reported moderate EE and DP levels, and low levels of PA. This may be attributed to several factors including poor income, comparable workload, and frequency of challenging cases when compared to licensed practitioners in our study. Our findings, together with those from previous research, emphasize that burnout has detrimental impacts on the personal and professional lives of perioperative care teams, resulting not only in mental health challenges but also physical symptoms and a decline in career, job, and workplace satisfaction.

Increasing evidence suggests that working night shifts is associated with a high prevalence of burnout [33]. The present study found that working night shifts exposed the respondents to a high level of DP. Our finding is consistent with a previous study suggesting that working extended hours in night shifts is associated with high EE and DP and low PA among anesthesiologists, anesthesia technicians, and intensive care unit (ICU) nurses [25]. This may be explained by the vulnerability of night shift workers to poor quality of life when compared to day shift workers. This is because night shift workers tend to experience reduced job satisfaction, adversely affected social and family life, and poor sleep quality [34]. Notably, we found that working night shifts is positively and independently associated with PA. These data contrast previous evidence [25]. It is maybe attributed to the younger population and single relationship status found in our study, indicating that they have less responsibilities toward family. In addition, it is possible that the reduced intensity of work and lower levels of interaction with the multidisciplinary teams during night shifts compared to day shifts may further explain our results [35]. Another contributing factor in our study to EE was the younger age. This is consistent with previous research showing that EE was associated with younger anesthesiologists [20,36,37]. These findings are supported by the fact that older healthcare providers have higher resilience, better work–life balance, and seeing positives when compared to younger healthcare providers [38].

Elevated stress levels and burnout are frequently linked, with constant stress leading to fatigue and emotional distress, particularly in high-demanding hospital areas like the operating theater [39]. Our findings show a significant relationship between elevated stress levels and all three principal dimensions of burnout. Emotional exhaustion, a fundamental aspect of burnout, is the sense of being emotionally drained and lacking the ability to fulfill professional requirements. Previous research reported high levels of EE among a heterogeneous group of anesthesia providers, including anesthesiologists, residents, and CRNAs [40]. Our study demonstrates a significant and independent association between EE, DP, and PA with elevated stress levels. Furthermore, we noted that respondents who had high EE and DP also reported the highest level of stress, indicating that chronic stress may be a primary component contributing to overall burnout among the anesthesia support personnel.

The findings of this study provided insights into the emotional exhaustion, depersonalization, lack of personal accomplishment, and levels of stress of this important group of the perioperative team in Saudi Arabia. These findings indicate how providing forms of accessible mental support services is advisable to improve the emotional wellbeing and to mitigate stress.

### Strengths and Limitations

The present study was strengthened by addressing a relatively overlooked topic among a group of perioperative healthcare providers. Another strength of the study is the use of a valid and reliable burnout instrument, namely MBI-HSS. However, it is also important to acknowledge that this research presents several limitations. The relatively small sample size, non-random sampling method, and single-center focus of this study may not fully capture the prevalence of burnout among the anesthesia technologists, technicians, and trainees in Saudi Arabia, leading to limited generalizability of the results. Furthermore, the method of distributing the questionnaire could lead to selection bias. Another limitation of the current study is the use of a single-item measure for assessing stress. Future research should consider employing validated, multidimensional tools to provide a more comprehensive evaluation of occupational stress. Lastly, certain variables, such as nationality and work experience, were not included in our analysis but may have influenced the levels of stress and burnout observed in our sample. Furthermore, it is important to note that a significant proportion of the workforce in Saudi Arabia consists of foreign workers. Therefore, future studies should consider incorporating nationality to ensure a more comprehensive understanding of occupational stress and burnout across the diverse workforce populations in Saudi Arabia.

## 5. Conclusions

In conclusion, the major findings of this study indicated that the burnout rate among anesthesia technologists, technicians, and trainees ranges from moderate to high, with stress identified as an independent factor of all three subscales of burnout. The solution to the issue is not simple and requires multiple steps. While many studies suggest personal strategies to encounter burnout and ensure balance and resilience for anesthesia technologists, the prevalence of burnout remains high. Therefore, it is important to encourage changes at both the policy and organizational levels. Implementing changes at the institutional, organizational, and national levels can bring about the desired change. These changes can be and are not limited to the following: defining optimal workload, improving efficiency, decreasing the administrative load, and providing a culture of compassion, engagement, and support, all of which are deeply needed to reduce the consequences of burnout for both the healthcare provider and the patient’s care. Furthermore, more research is needed at the national level to investigate the effectiveness of current wellbeing and mental health promotion centers.

## Figures and Tables

**Table 1:** Characteristics of the study respondents.

Variable	All (n = 89)	Anesthesia Technologists/Technicians (n = 58)	Trainees (n = 31)	*p* Value
Age	24.0 (4.0)	26.0 (6)	22 (1)	<0.001
Gender, male	68 (76.4)	52 (90%)	16 (52%)	<0.001
Marital status, single	71 (79.8)	40 (69%)	31 (100%)	0.001
Smoking, yes	32 (36.0)	27 (47%)	5 (16%)	0.009
Income				
Less than SAR 6000	33 (37.1)	3 (5.2%)	30 (97%)	<0.001
SAR 6000–9000	20 (22.5)	19 (33%)	1 (3.2%)	
SAR 9000–12,000	24 (27.0)	24 (41%)	0 (0)	
SAR 12,000–16,000	8 (9.0)	8 (14%)	0 (0)	
More than SAR 16,000	4 (4.5)	4 (6.9%)	0 (0)	
Number of cases per week	4.0 (2.0)	4.0 (2.0)	4.0 (2.0)	0.1
Frequency of challenging cases				
Once per month	15 (16.9)	10 (17%)	5 (16%)	>0.9
Once per week	28 (31.5)	18 (31%)	10 (32%)	
2–3 times per week	32 (36.0)	20 (34%)	12 (39%)	
Almost everyday	8 (9.0)	5 (8.6%)	3 (9.7%)	
Almost none	6 (6.7)	5 (8.6%)	1 (3.2%)	
Night shift, yes	41 (46.1)	40 (69%)	1 (3.2%)	<0.001
Total emotional exhaustion score	23 (22)	23 (21)	24 (25)	0.8
Total depersonalization score	6 (9)	6 (10)	6 (7)	0.4
Total personal accomplishment score	31 (18)	32 (20)	29 (16)	0.3
Stress level	7 (4)	7 (4)	6 (3)	0.5

Categorical data are presented as n (%) and continuous data are presented as median (IQR).

**Table 2:** Level of burnout according to subscales.

Level of Burnout	Emotional Exhaustion	Depersonalization	Personal Accomplishment
High	34 (38.2)	25 (28.1)	52 (58.4)
Moderate	21 (23.6)	25 (28.1)	14 (15.7)
Low	34 (38.2)	39 (43.8)	23 (25.8)

Data are presented as n (%).

**Table 3:** Comparison of stress levels based on emotional exhaustion, depersonalization, and personal accomplishment level.

Burnout Subscale	Stress Level	*p*-Value
Emotional exhaustion		**<0.001**
High	8.0 (2.0)	
Moderate	6.0 (3.0)	
Low	5.5 (4.0)	
Depersonalization		**0.015**
High	8.0 (2.0)	
Moderate	6.0 (3.0)	
Low	6.0 (5.0)	
Personal accomplishment		**0.048**
High	8.0 (3.0)	
Moderate	7.0 (3.25)	
Low	6.0 (4.0)	

Note: bold indicates statistically significant at <0.05.

**Table 4:** Univariable and multivariable linear regression analysis of factors associated with emotional exhaustion.

	Univariate Analysis Beta [95% CI]	*p*-Value	Multivariate Analysis Beta [95% CI]	*p*-Value
Age	−0.50 [−0.94, −0.05]	**0.029**	−0.58 [−0.95, −0.20]	**0.003**
Gender		0.14		
Male	—		—	0.6
Female	5.0 [−1.7, 12]		1.7 [−3.8, 7.2]	
Smoking		>0.9		
No	—			
Yes	−0.09 [−6.0, 5.8]			
Marital status		0.064		
Single	—			
Married	−6.6 [−14, 0.39]			
Frequency challenging cases		**0.010**		0.11
Once per month	—		—	
Once per week	2.0 [−6.2, 10]		4.9 [−2.0, 12]	
2–3 times per week	8.0 [0.09, 16]		7.4 [0.71, 14]	
Almost everyday	−8.9 [−20, 2.2]		−1.7 [−12, 8.2]	
Almost none	−1.6 [−14, 11]		6.5 [−4.2, 17]	
Night shift		0.14		
No	—			
Yes	4.2 [−1.4, 9.9]			
Income		>0.9		
Less than SAR 6000	—			
SAR 6000–9000	−1.1 [−8.8, 6.6]			
SAR 9001–12,000	−0.14 [−7.4, 7.2]			
SAR 12,001–16,000	−2.1 [−13, 8.7]			
More than SAR 16,000	−6.9 [−21, 7.5]			
Stress level	3.3 [2.1, 4.4]	**<0.001**	3.3 [2.1, 4.5]	**<0.001**

Note: bold indicates statistically significant at <0.05.

**Table 5:** Predictors of depersonalization.

	Univariate Analysis Beta [95% CI]	*p*-Value	Multivariate Analysis Beta [95% CI]	*p*-Value
Age	−0.04 [−0.26,0.18]	0.7		
Gender		0.7		0.5
Male	—		—	
Female	−0.52 [−3.7, 2.7]		1.2 [−2.2, 4.6]	
Smoking		** 0.032 **		0.13
No	—		—	
Yes	3.0 [0.26, 5.8]		2.2 [−0.68, 5.0]	
Marital status		0.7		
Single	—			
Married	−0.75 [−4.1, 2.6]			
Frequency challenging cases		0.3		
Once per month	—			
Once per week	−4.2 [−8.2, −0.11]			
2–3 times per week	−3.2 [−7.2, 0.75]			
Almost everyday	−5.2 [−11, 0.37]			
Almost none	−3.7 [−9.9, 2.4]			
Night shift		**0.016**		**0.024**
No	—		—	
Yes	3.3 [0.61, 5.9]		3.3 [0.44, 6.1]	
Income		0.7		
Less than SAR 6000	—			
SAR 6000–9000	2.6 [−1.0, 6.3]			
SAR 9001–12,000	1.2 [−2.2, 4.7]			
SAR 12,001–16,000	1.8 [−3.3, 6.9]			
More than SAR 16,000	1.5 [−5.3, 8.4]			
Stress level	0.74 [0.13, 1.4]	**0.018**	0.73 [0.13, 1.3]	**0.017**

Note: bold indicates statistically significant at <0.05.

**Table 6:** Predictors of personal accomplishment.

	Univariate Analysis Beta [95% CI]	*p*-Value	Multivariate Analysis Beta [95% CI]	*p*-Value
Age	−0.21 [−0.61,0.19]	0.3		
Gender		0.6		0.068
Male	—		—	
Female	1.6 [−4.3, 7.6]		5.8 [−0.42, 12]	
Smoking		0.8		
No	—			
Yes	0.63 [−4.6, 5.9]			
Marital status		0.7		
Single	—			
Married	1.1 [−5.2, 7.4]			
Frequency challenging cases		0.8		
Once per month	—			
Once per week	0.94 [−6.7, 8.6]			
2–3 times per week	2.2 [−5.3, 9.8]			
Almost everyday	3.7 [−6.8, 14]			
Almost none	6.9 [−4.7, 18]			
Night shift		**0.048**		**0.014**
No	—		—	
Yes	5.0 [0.04, 10]		6.6 [1.4, 12]	
Income		0.4		
Less than SAR 6000	—			
SAR 6000–9000	5.5 [−1.2, 12]			
SAR 9001–12,000	−1.0 [−7.4, 5.3]			
SAR 12,001–16,000	1.7 [−7.7, 11]			
More than SAR 16,000	5.1 [−7.5, 18]			
Stress level	−1.2 [−2.3, −0.03]	**0.043**	−1.3 [−2.4, −0.12]	**0.03**

Note: bold indicates statistically significant at <0.05.

## Data Availability

The data that support the findings of this study are available on reasonable request from the corresponding author, but restrictions apply due to privacy and ethical restrictions.
